# “We Are the Sons of Our Own Deeds”: Comparing Skeletal Health and Frailty Indices in Deceased Individuals Across 2000 Years of Milanese History

**DOI:** 10.1002/ajpa.70025

**Published:** 2025-03-19

**Authors:** Petrosino, L. Biehler‐Gomez, K. E. Marklein, M. Mondellini, C. Moro, M. Mattia, A. M. Fedeli, C. Cattaneo

**Affiliations:** ^1^ Laboratorio di Antropologia e Odontologia Forense (LABANOF), Dipartimento di Scienze Biomediche per la Salute Università Degli Studi di Milano Milan Italy; ^2^ Department of Anthropology University of Louisville Louisville Kentucky USA; ^3^ Center for Archaeology and Cultural Heritage University of Louisville Louisville Kentucky USA; ^4^ Soprintendenza Archeologia Belle Arti e Paesaggio per la Città Metropolitana di Milano Milan Italy

**Keywords:** bioarchaeology, frailty, frailty index, paleopathology, stress markers

## Abstract

**Objectives:**

In bioarchaeology, the concepts of resilience and frailty, and their quantification through indices, have gathered significant attention. This study is the first to apply, evaluate, and compare skeletal frailty indices and aims to trace frailty over time while identifying methodological challenges in their use on a sample representative of urban Milan's history.

**Materials and Methods:**

Two‐hundred fifty individuals from five historical periods over 2000 years in urban Milan, equally represented by estimated males and females, were analyzed. Three skeletal frailty indices were applied—the “Health Index” GHHP, “Skeletal Frailty Index” (SFI), and “Biological Index of Frailty” (BIF)—and their diachronic variations interpreted. Index values were compared to each other through Spearman's correlations, and frailty values were assessed by periods (overall and by estimated sex) and by estimated sex through ANOVA and General Linear Models.

**Results:**

Diachronic analyses revealed a gradual increase in frailty from the Roman era to the Late Middle Ages, which then progressively decreased, corroborating historical sources. While all methods identified the Late Middle Ages sample as the frailest, discrepancies arose when defining the least frail group, especially when considering estimated biological sex and age variables.

**Discussion:**

Our study found practical and conceptual limitations in the GHHP. Most noticeably, criteria for GHHP and SFI limited sample size (and consequently) representation, while the more inclusive BIF proved overly permissive, allowing direct comparisons between skeletons with differential preservation. This study highlights common challenges and prospects, defines common criteria to standardize methodologies, and further investigates the relevance of stress markers in relation to frailty.

## ABSTRACT (ITALIAN)

**Obiettivi:**

Nell'ambito della bioarcheologia, i concetti di resilienza e fragilità, e la loro quantificazione attraverso appositi indici, hanno destato notevole interesse. Questo studio è il primo ad applicare, valutare e confrontare gli indici scheletrici di fragilità, con l'intento di tracciare l'evoluzione del concetto di fragilità nel tempo e identificare le sfide metodologiche nel loro utilizzo su un campione rappresentativo della storia urbana di Milano.

**Materiali e Metodi:**

Sono stati analizzati duecentocinquanta individui appartenenti a cinque epoche storiche lungo 2000 anni nella Milano urbana, equamente rappresentati da maschi e femmine. Sono stati applicati tre indici di fragilità scheletrica—l’”Health Index” (GHHP), lo “Skeletal Frailty Index” (SFI) e il “Biological Index of Frailty” (BIF) — le cui variazioni diacroniche sono state interpretate. I valori degli indici sono stati confrontati tra loro mediante correlazioni di Spearman, e i valori di fragilità valutati per periodi (complessivamente e per sesso stimato) e per sesso stimato attraverso ANOVA e Modelli Lineari Generalizzati.

**Risultati:**

Le analisi diacroniche hanno rivelato un graduale incremento della fragilità dall'epoca Romana al Tardo Medioevo, seguito da una progressiva diminuzione, confermando le fonti storiche. Sebbene tutti i metodi abbiano identificato il campione del Tardo Medioevo come il più fragile, sono emerse discrepanze nella definizione del gruppo meno fragile, in particolare con l'introduzione delle variabili sesso biologico stimato ed età alla morte.

**Discussione:**

Lo studio ha evidenziato limiti pratici e concettuali nel GHHP. Fra questi, i criteri di GHHP e SFI hanno ridotto la dimensione del campione (e conseguentemente la rappresentatività), mentre il più inclusivo BIF si è rivelato eccessivamente permissivo nel consentire il confronto diretto tra scheletri con diverso grado di conservazione. Questo lavoro mette in luce le sfide e le prospettive comuni, definisce criteri condivisi per standardizzare le metodologie e indaga ulteriormente la rilevanza dei marcatori di stress in relazione alla fragilità.

## 1 Introduction

The World Health Organization defines “health” as “a state of complete physical, mental, and social well‐being, distinct from the mere absence of disease or infirmity” (World Health Organization 1946). Although understanding health in past populations is a critical theme and research interest in bioarchaeology, inferring health status of long past populations using deceased individuals as proxies still requires reconciling conceptual elements particular to living populations with those of past populations. However, leveraging a paleopathological perspective that incorporates the osteological paradox, recent bioarchaeological studies have made theoretical and methodological strides in health and stress interpreting skeletal biomarkers and operationalizing concepts of resilience and frailty (e.g., DeWitte and Stojanowski 2015; Abete et al. 2017; Kyle et al. 2018; Frazier 2022; Zedda et al. 2022).

In their commentary on the Osteological Paradox (1992), Wood and colleagues clarified several misunderstood aspects of the discussion, such as Cohen's proposal of “decrepitude” as a more suitable alternative to “frailty.” While framing frailty as decrepitude is constructive for linking frailty in the past with frailty—and frailty phenotypes and indices (Fried et al. 2001)—today, much of the osteological paradox focused on risk of mortality. This focus on the “risk aspect” of frailty overlooks resilience and the adaptive responses to stressful experiences, represented an individual's ability to withstand and survive stress events (Holling 1973; Temple and Stojanowski 2018).

Despite this and numerous other clarifications, the concept of frailty remains challenging, and considerable emphasis has been placed on using the presence of stress markers as a direct indicator of health, which oftentimes oversimplifies the issue to syllogistic relationships, for example, presence of skeletal lesions equals poor health and absence equals good health (DeWitte and Stojanowski 2015). Only later did a greater awareness of the frailty‐resilience dualism emerge, and a better understanding of it was achieved, defined as “a fluctuating physical state based on exposures to and survival from diseases and other stressors” (Marklein et al. 2016), or as “the state of physiological stress that an individual suffered during [their] life and that caused [their] susceptibility to diseases and death” (Zedda et al. 2021). Resilient individuals live through hardships, although cumulative frailty following these hardships results in increased risk of disease and death (Lucas 1998; Waterland and Michels 2007; Hanson and Gluckman 2008; Gowland 2015; Larsen 2015).

The development of skeletal health and frailty indices began in the late 19th century with methods developed by forensic and rehabilitation physicians for granting pensions to American Civil War veterans, based on individual disability assessments (Goodman and Martin 2002). However, these methods were inadequate for application to archaeological contexts as they used underrepresentative samples drawn from military draft records and were based on early conceptualizations of stress (Steckel, Sciulli, et al. 2002). *Paleopathology at the Origins of Agriculture* (Armelagos and Cohen 1984) codified interest in the health status of past populations—and its applicability to contemporary issues in health—and proposed that population growth before the Neolithic period set the stage for nutritional deficiencies spurred by the development and intensification of agriculture. Later, *The Backbone of History* (Steckel, Rose, et al. 2002) investigated the history of health in the global Western world by assembling specialists from various disciplines and analyzing over 12,500 skeletons. These foundational publications inspired current population studies and health and stress models, coding biomarkers of biological stress (indicative of “health deficits”) and quantifying the quality of life at both individual and population levels; these methods allowed diachronic and synchronic comparisons between populations, even when geographically and temporally remote.

Current bioarchaeological literature proposes three methods for operationalizing cumulative stress and/or frailty: Steckel and Rose's (2002) Health Index, Marklein and colleagues' Skeletal Frailty Index (Marklein et al. 2016; Marklein and Crews 2017), and Zedda and colleagues' Biological Index of Frailty (Zedda et al. 2021). This paper will document the application, advantages, limitations, and diachronic trends of frailty offered by these methods when applied to 250 skeletons representative of 2000 years of Milanese history (50 skeletons per historical period), drawn from a human osteological collection in Milan, with the hope of providing useful insight for future investigations. It is our intention to (1) assess the comparability of these indices; (2) scrutinize the informativeness of each indexical approach; and (3) evaluate diachronic patterns in frailty in Milan through these differing but potentially complementary methods.

## 2 Materials and Methods

The CAL (Anthropological Collection of the LABANOF—*Collezione Antropologica LABANOF*) is an osteological collection including over 7000 deceased individuals, originating from archaeological sites within Milan and the region of Lombardy, as well as contemporary funerary contexts of unclaimed individuals from Milanese cemeteries.

From this larger collection, 250 adult individuals were selected, evenly represented between males and females, resulting in 25 estimated females and 25 estimated males per historical period. Estimated age and sex distributions of this sample are presented in Table 1. Five historical periods, spanning two millennia of Milanese history, were included: Roman period (1st–5th century CE), Early Middle Ages (6th–10th century CE), Late Middle Ages (11th–15th century CE), Modern period (16th–18th century CE), and Contemporary period (19th–21st century CE).

**TABLE 1:** Estimated age distribution of Milanese individuals included in this study by period and estimated sex (F = estimated likely female, M = estimated likely male).

	Roman era *n*/*N* (%)	Early middle ages *n*/*N* (%)	Late middle ages *n*/*N* (%)	Modern era n/*N* (%)	Contemporary era *n*/*N* (%)
16–20 years	5/50 (10%)	5/50 (10%)	6/50 (12%)	4/50 (8%)	0/50 (0%)
21–30 years	12/50 (24%)	13/50 (26%)	11/50 (22%)	7/50 (14%)	5/50 (10%)
31–45 years	22/50 (44%)	15/50 (30%)	15/50 (30%)	15/50 (30%)	3/50 (6%)
46–60 years	10/50 (20%)	17/50 (34%)	17/50 (34%)	21/50 (42%)	6/50 (12%)
61–80 years	1/50 (2%)	0/50 (0%)	1/50 (2%)	3/50 (6%)	20/50 (40%)
> 80 years	0/50 (0%)	0/50 (0%)	0/50 (0%)	0/50 (0%)	16/50 (32%)

	F	M	F	M	F	M	F	M	F	M
16–20 years	4	1	2	3	4	2	3	1	0	0
21–30 years	10	2	6	7	4	7	4	3	2	3
31–45 years	8	14	9	6	7	8	9	6	0	3
46–60 years	3	7	8	9	9	8	8	13	1	5
61–80 years	0	1	0	0	1	0	1	2	10	20
> 80 years	0	0	0	0	0	0	0	0	12	4

All skeletal remains derive from the same urban context, that is, the city of Milan. Historical records and archaeological excavation data indicate that these individuals belong to the lower (rarely middle) socioeconomic strata of Milanese society. The osteological material employed in this study, dated through stratigraphic analysis, interpretation of grave goods or contextual artifacts, as well as radiocarbon dating of bone samples (Biehler‐Gomez, del Bo, et al. 2023), originates from five burial sites.

- The archaeological site of the *Università Cattolica*, with 600 skeletons dated to Imperial Rome/Late Antiquity (corresponding to the Roman period for our study) (Biehler‐Gomez et al. 2022; Istituto Centrale per l'Archeologia (ICA) 2022);
- The emergency excavations of the M4 metropolitan line in the area of the Basilica of Sant'Ambrogio, encompassing 93 tombs stratigraphically dated from the 1st to the 15th century CE. From this context, both Early Middle Ages and Late Middle Ages individuals were included (Sannazaro et al. 2009; Biehler‐Gomez et al. 2022);
- The emergency excavations of the M4 metropolitan line in the area of San Vittorecomprised 96 burials from the 3rd century AD to the 17th century CE, from which only individuals found in anatomical connection dating to the Late Middle Ages were selected;
- The collective burials of 260 individuals unearthed at the corner of *Viale Sabotino* and the remnants of the Spanish walls near *Porta Romana* were found in anatomical connection and attributed to hospital or epidemic deaths of the 17th century CE (Caruso et al. 2013) (pertaining to the Modern period of the sample); and
- The cemetery collection of the CAL, consisting of 2127 unclaimed skeletal remains of individuals deceased in the second half of the 20th century in the principal cemeteries of Milan, represents the contemporary portion of the sample (Cattaneo et al. 2018).

*Ethics statement*. An agreement with the *Sopraintendenza Archeologia, Belle Arti e Paesaggio della Lombardia* (the regional body of the Italian Ministry of Cultural Heritage) laid the ethical and scientific guidelines under which the analysis of archaeological remains was conducted. The study of anonymized contemporary remains required no informed consent and was authorized and regulated by Article 43 of the Presidential Decree of the Italian Republic (DPR) n.285, dated September 10, 1990, within the framework of the National Police Mortuary Regulation and in collaboration with the Health Territorial Agency of Milan. All procedures complied with Italian legislation, as well as institutional policies and regulations. These individuals represent a subgroup of the larger DOMINA project, which focuses on the lived experiences of females in historic Milan and has continued local and global support.^1^ ^,^ ^2^

As post‐mortem degradation may preclude a complete biological profile and detection of skeletal biomarkers (Cattaneo and Grandi 2004), only the most intact and well‐preserved skeletons were selected. To do so, excavation photographic records were used as references. Moreover, as the study sample was meant to be equally representative of both male and female individuals, a lower age limit of 16 years was set for the selection of individuals. This age typically coincides with the complete fusion of the coxal bones, allowing for reliable sex estimation using Phenice (1969), Klales et al. (2012), Walker (2008) and Spradley and Jantz (2011). Regarding estimated age‐at‐death, estimations were performed based on dental age (Mincer et al. 1993; Kvaal and Solheim 1995; AlQahtani et al. 2010), skeletal growth and development (Scheuer and Black 2004), and changes to sternal rib ends, iliac auricular surfaces, and pubic symphyses (İşcan et al. 1984; Lovejoy et al. 1985; Brooks and Suchey 1990; Rougé‐Maillart et al. 2009). Individuals were subsequently categorized into the following age groups: 16–20 years; 21–30 years; 31–45 years; 46–60 years; 61–80 years; > 80 years.

### 2.1 Skeletal Indexes of Health and Frailty

In order to assess the three indices of health and frailty, we calculated values according to the methods outlined in Steckel and Rose (2002), Marklein et al. (2016), and Zedda et al. (2021). Consequently, for each of the 250 individuals, we obtained three index scores. However, as these scores represent different scales of poor/good health or frailty—0–100 (least to most healthy) range for the Health Index, 0–6 (least to most frail) for the SFI, and 0–100 (least to most frail) for the BIF—we converted all index scores to a 0–100 scale. For example, a Health Index score of 40 had a converted score of 60, and an SFI of 1 (1 of 6) had a 16.7 score (see below). Such a conversion enabled comparison of index values between methods.

#### 2.1.1 Global History of Health Project: Health Index (Steckel and Rose 2002)

Described in *The Backbone of History: Health and Nutrition in the Western Hemisphere* (Steckel and Rose 2002), Steckel and Rose's index was the first cumulative health approach proposed in paleopathological literature. Using 12,520 skeletons over a span of 7000 years, from 218 sites across North, Central, and South America, organized into 65 groups based on chronological and ecological similarities, this method analyzed seven skeletal biomarkers (Table 2) and implemented synthetic reference populations (Thompson et al. 1967). The authors defined the index as “the sum of the quality‐adjusted life years in which mortality experience is defined by a Model West level 4 life table” (Steckel, Rose, et al. 2002, 147).

**TABLE 2:** List of stress markers for each method considered in the present study.

GHHP (0–100) Steckel and Rose (2002)	SFI (0–6) Marklein et al. (2016)‐ Marklein and Crews (2017)	BIF (0–9) Zedda et al. (2021)
Low stature	Periodontal disease	Low stature
Enamel hypoplasia	Linear enamel hypoplasia	Low body mass
Anemia	Intervertebral disk disease	Linear enamel hypoplasia
Infections	Periosteal reactions	Peridontal disease
Degenerative joint disease	Fracture	Periostitis
Dental health	Osteoarthrosis	Cribra orbitalia
Trauma		Porotic hyperostosis
		Rickets/osteomalacia
		Osteoarthrosis and other joint disease
		Vertebral disease
		Trauma

*Note:* Biomarkers were selected for each index to be as inclusive of conditions (e.g., childhood stress, nutritional stress, infection, disease, degenerative wear, and trauma/fracture).

Each biomarker is assessed on a scale of 0–100, where 0 represents the worst possible health and 100 represents the best possible health or at least the absence of lesions or signs of biological stress. Scores are assigned as continuous or discrete values depending on the attribute considered. For example, stature is first calculated using regression formulas and then scored as 100 if the value meets the modern standard, or 0 if it falls below. On the other hand, DJD (degenerative joint disease) is first evaluated per major joint groups. Each group is given a score based on the most severe manifestation of the marker from either the right or left side, using a scale provided by the authors (i.e., hip and knee, evaluated as a single unit, is scored as follows: 0, 25, 50, 75, and 100). Lastly, the final stress marker score corresponds to the worst score recorded among all areas.

The method ultimately provides a measure of site‐specific health status, using life expectancy and quality estimates in the form of “accumulated life years” as predictive tools. However, the goal of this research is not to predict life expectancy of Milanese populations but to investigate the evolution of the (modern concept of) frailty by testing the applicability of the three available indices in literature. Thus, the “accumulated life years” and “overall quality score as a percentage of the maximum” were excluded from this study, focusing instead on the evaluation of stress markers. The health index was derived by calculating the arithmetic mean of the stress marker scores, provided all were assessable according to the authors' guidelines. This approach removed a notably cumbersome portion of the original method (Marklein and Crews 2017; Hubbe et al. 2018), eliminating many conceptual assumptions that, as described by Woods and colleagues (Wood et al. 1992) in the “osteological paradox,” would compromise the interpretation of health from osteological material. Additionally, the “dental health” marker was calculated solely by evaluating the “abscesses” component, disregarding the “completeness” component, which returned disproportionately high or even negative values. These figures were difficult to interpret and would have skewed the final index result toward near‐zero values, thus underestimating frailty.

#### 2.1.2 Skeletal Frailty Index (SFI) (Marklein et al. 2016; Marklein and Crews 2017)

Marklein and colleagues' method (Marklein et al. 2016), and the updated version (Marklein and Crews 2017; Tuggle et al. 2021), derives from clinical and gerontological concepts of frailty in the living and represents a significant shift from the health indices; the SFI does not reference life expectancy calculations and is considerably simplified, generally requiring only “presence/absence” scoring for indicated stress markers representative of four general stress categories: trauma, nutrition/disease, physical activity, and growth disruptions.

The original SFI represents the sum of frailty scores assigned and ranges from 0 to 13, with a higher index corresponding to greater frailty. SFI scores are initially tabulated by individual and then compared with the specific population.

Although the index proves to be statistically robust, the method requires well‐preserved skeletal material, since the absence of even one element prevents the calculation of the index. As a result, out of the 976 skeletons the authors selected from the Museum of London (MoL) open‐access Wellcome Osteological Research Database (WORD), only 134 were well‐preserved enough for inclusion in the original study sample (Marklein et al. 2016). Thus, a more parsimonious index was developed based on fewer parameters (Marklein and Crews 2017). In this study, we applied the 6‐biomarker SFI (Marklein and Crews 2017), as it expands applicability and representativeness while maintaining statistical robustness, albeit being less informative than the original method (Table 2). Using presence/absence and activity (periosteal lesions), individuals were scored on a 0 (low frailty) to 6 (high frailty) scale.

#### 2.1.3 Biological Index of Frailty (BIF) (Zedda et al. 2021)

The BIF was developed using the same monastic and non‐monastic skeletal assemblage from MoL's WORD used to develop the SFI. The main innovations introduced in this methodology are twofold: (1) it can be applied to incomplete skeletons, a fact ensured by calculating the index through a weighted mean of the scorings and requiring a minimum of only three stress biomarkers per skeleton; and (2) it considers the severity or remodeling of lesions for specific conditions.

Each biological frailty marker (Table 2) is tied to a weight, whose assignment is based on the hypothesis that individuals who died prematurely would show biomarkers contributing more significantly to individual frailty. The authors used a Logit model to estimate the “odds ratios” (OR and 95% confidence interval) of premature death for each biomarker, defining “premature death” using life tables (Chamberlain 2006; Mallegni and Lippi 2009) to calculate the average life expectancy of the studied necropolis. As with the SFI, the BIF is developed from and for a specific population and the distribution of biomarkers therein.

To conclude population frailty assessment, the authors provided index interpretations: values of 0–21 indicating low frailty, 21–53 indicating medium frailty, and 53–100 indicating high frailty.

### 2.2 Statistical Analyses

Contextual (site, period, estimated sex, and estimated age) and indexical (GHHP, SFI, and BIF scores) data are included in the Supporting Information Table. For diachronic comparisons of frailty scores, univariate ANOVA, Bonferroni post hoc test, and general linear models were utilized. In all assessments, frailty values were set as the dependent variable, while categorical factors (time period, estimated sex, and age group) were considered independent variables.

Additionally, Spearman's correlations were employed on subsamples of individuals who could be scored for health or frailty according to GHHP, SFI, and BIF criteria to assess the comparability of these indices. For these tests, 114 individuals had scores for GHHP and SFI criteria, 119 individuals for GHHP and BIF criteria, and 140 individuals for SFI and BIF criteria. All statistical analyses were conducted in SPSS 29; statistical significance was set as *p* ≤ 0.05 and approaching significance 0.05 < *p* ≤ 0.10.

## 3 Results

For this project, we (1) tested the applicability and comparability of three indices utilized in bioarchaeological research on 250 individuals from the CAL collection; and (2) examined changes in these health and frailty values over time in Milan.

### 3.1 Applicability and Comparability of Health and Frailty Indices

**FIGURE 1:**
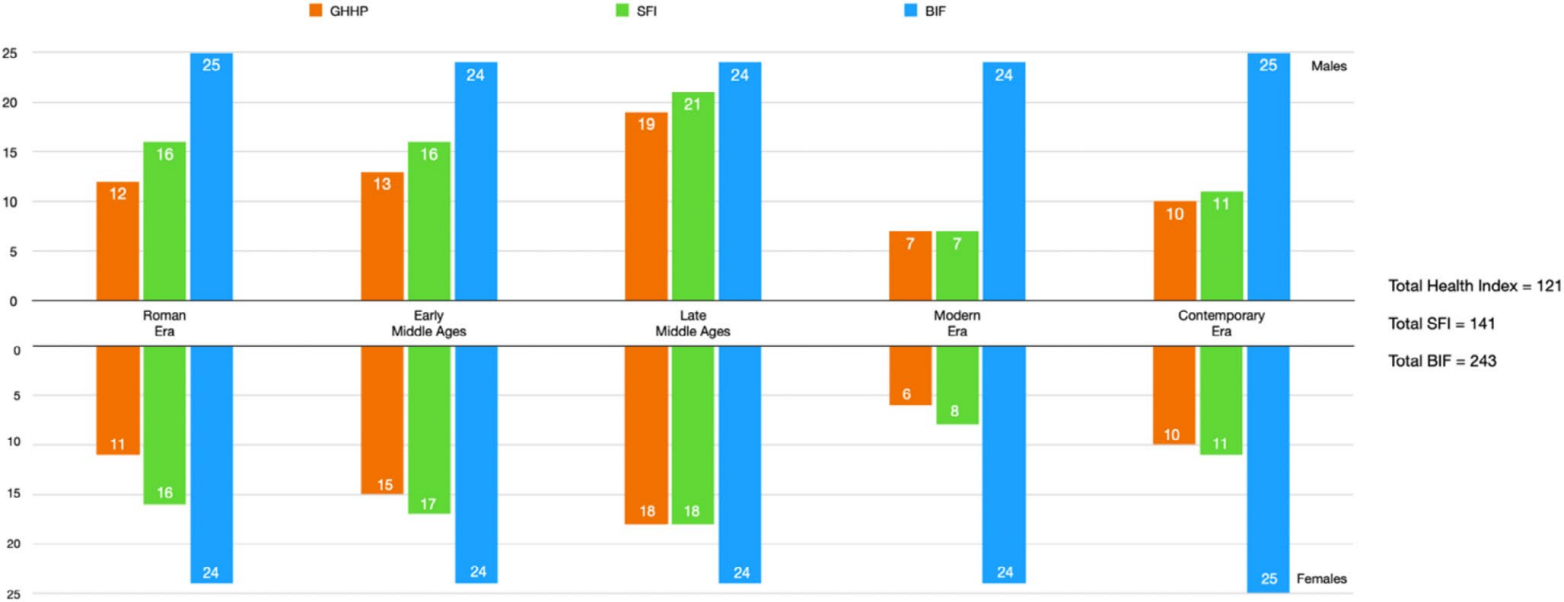
Applicability of the three frailty indices (values are shown as case numbers).

When applied to the 250 individuals in CAL, indices yielded different samples. Only 121 individuals (48% of the original sample) were scorable for the Health Index (Steckel and Rose 2002); 121 individuals (48%) were scorable for the original SFI, and 141 individuals (56%) were scorable for the modified SFI (Marklein and Crews 2017); and 243 individuals (97%) were scorable for the BIF criteria (Zedda et al. 2021) (Figure 1). Of the five periods studied, the Late Middle Ages had the most individuals scorable for all three indices and thus the most representative sample. In contrast, the Modern Era was the least represented: the Health Index was applicable to 26%, the SFI was applicable to 30%, and the BIF was applicable to 97% of the 250‐individual sample. The higher applicability of BIF to this sample is expected, as the BIF was developed to increase comparative sample size.

GHHP, SFI, and BIF scores were analyzed through Spearman's correlations to assess the comparability of indices (Table 3). Significant correlations were observed between all indices; however, only the correlation between GHHP and SFI values is considered high (rho = 0.531). The correlations between GHHP and BIF (rho = 0.437) and SFI and BIF (rho = 0.474) exhibit a medium correlation.

**TABLE 3:** Spearman's correlations for GHHP, SFI, and BIF values.

		GHHP	SFI	BIF
GHHP	rho		0.531**	0.474**
	*p*		< 0.001	< 0.001
	*N*		114	119
SFI	Rho			0.437**
	*p*			< 0.001
	*N*			140
BIF	Rho			
	*p*			
	*N*			

*Note:* *p ≤ 0.10, **p value ≤ 0.05.

### 3.2 Trends of Frailty in Diachronic Milan

In addition to comparisons through Spearman's correlations, health and frailty index values were compared diachronically. Distributions of frailty according to methodological approach by period, estimated sex, and estimated age are presented in Table 4. Generally, all methods yielded frailty values that increased from the Roman Era to the Late Middle Ages, followed by a decrease in values into the Contemporary Era. For all methods, the Late Middle Ages yielded the highest frailty values. However, the lowest frailty averages differed based on the selected method: the Modern Era sample (according to the Health Index), the Roman Era sample (according to the SFI), and the Contemporary Era sample (according to the BIF).

**TABLE 4:** Average indices by period, estimated sex, and age group.

Biological profile		Roman era			Early middle ages			Late middle ages			Modern era			Contemporary era		
Age group	Biological sex	Health index	SFI	BIF	Health index	SFI	BIF	Health index	SFI	BIF	Health index	SFI	BIF	Health index	SFI	BIF
16–20	M	—	50.00	57.14	57.14	66.67	29.49	39.93	41.67	59.72	21.43	16.67	26.32	—	—	—
	F	14.29	8.33	14.85	28.57	33.33	16.67	39.53	44.44	48.68	21.43	16.67	35.71	—	—	—
21–30	M	—	16.67	45.00	19.09	30.95	32.54	32.76	42.86	34.57	10.21	33.33	37.94	27.05	50.00	32.38
	F	22.24	22.22	18.64	33.96	29.17	32.20	10.71	38.89	46.62	—	—	25.16	19.14	16.67	19.90
31–45	M	32.53	37.50	33.44	9.34	8.33	42.25	34.79	54.76	36.07	—	83.33	41.11	0.00	0.00	17.42
	F	27.14	23.33	17.41	18.18	23.33	33.98	31.30	53.33	33.57	19.10	33.33	42.55	—	—	—
46–60	M	25.96	54.17	38.72	40.21	52.78	38.69	34.57	50.00	42.31	23.05	41.67	38.49	19.52	44.44	20.32
	F	16.79	50.00	26.71	36.79	57.14	33.39	25.23	47.22	29.82	10.14	38.89	28.69	4.86	16.67	23.81
61–80	M	32.14	66.67	30.00	40.21	52.78	38.69	—	—	—	9.57	—	21.88	22.48	44.44	24.63
	F	—	—	—	40.21	52.78	38.69	50.14	66.67	35.00	—	—	22.22	30.07	54.17	25.59
> 80	M	—	—	—	—	—	—	—	—	—	—	—	—	—	—	22.06
	F	—	—	—	—	—	—	—	—	—	—	—	—	21.43	45.83	23.80
Minimum value	M	25.96	16.67	30.00	9.34	8.33	29.49	32.76	41.67	34.57	9.57	16.67	21.88	0.00	0.00	17.42
	F	14.29	8.33	14.85	18.18	23.33	16.67	10.71	38.89	29.82	10.14	16.67	22.22	4.86	16.67	19.90
Maximum value	M	32.53	66.67	57.14	57.14	66.67	42.25	39.93	54.76	59.72	23.05	83.33	41.11	27.05	50.00	32.38
	F	27.14	50.00	26.71	40.21	57.14	38.69	50.14	66.67	48.68	21.43	38.89	42.55	30.07	54.17	25.59

*Note:* Minimum and maximum values are reported in the bottom rows.

Results from ANOVA showed significant differences in GHHP and BIF by period (Table 5), but these differences varied based on the method. For example, significantly lower frailty was observed between the Late Middle Ages and Modern Era according to GHHP measures, while significant increases in frailty were observed between the Early Middle Ages and Modern Era and significant decreases between the Late Middle Ages and Contemporary Era and between the Modern Era and Contemporary Era according to BIF measures.

**TABLE 5:** Results from ANOVA tests.

Dependent variable	Independent variable	Sig.
GHHP score	By period	0.004**
	Roman era and early middle ages	1.000
	Roman era and late middle ages	0.676
	Roman era and modern era	0.645
	Roman era and contemporary era	1.000
	Early middle ages and late middle ages	1.000
	Early middle ages and modern era	0.103
	Early middle ages and contemporary era	0.846
	Late middle ages and modern era	0.006**
	Late middle ages and contemporary era	0.064*
	Modern era and contemporary era	1.000
	By estimated sex	0.416
	By age	0.507
SFI score	By period	0.068*
	Roman era and early middle ages	1.000
	Roman era and late middle ages	0.040**
	Roman era and modern era	1.000
	Roman era and contemporary era	1.000
	Early middle ages and late middle ages	0.852
	Early middle ages and modern era	1.000
	Early middle ages and contemporary era	1.000
	Late middle ages and modern era	1.000
	Late middle ages and contemporary era	1.000
	Modern era and contemporary era	1.000
	By estimated sex	0.156
	By age	0.014**
	16–20 years and 21–30 years	1.000
	16–20 years and 31–45 years	1.000
	16–20 years and 46–60 years	0.893
	16–20 years and 61–80 years	1.000

	16–20 years and over 80 years	1.000
	21–30 years and 31–45 years	1.000
	21–30 years and 46–60 years	0.029**
	21–30 years and 61–80 years	0.276
	21–30 years and over 80 years	1.000
	31–45 years and 46–60 years	0.221
	31–45 years and 61–80 years	0.854
	31–45 years and over 80 years	1.000
	46–60 years and 61–80 years	1.000
	46–60 years and over 80 years	1.000
61–80 years and over 80 years	1.000
BIF score	By period	< 0.001**
	Roman era and early middle ages	0.927
	Roman era and late middle ages	0.066
	Roman era and modern era	0.394
	Roman era and contemporary era	1.000
	Early middle ages and late middle ages	1.000
	Early middle ages and modern era	1.000
	Early middle ages and contemporary era	0.046*
	Late middle ages and modern era	1.000
	Late middle ages and contemporary era	0.001**
	Modern era and contemporary era	0.013**
	By estimated sex	0.014**
	By age	0.133

*Note:* Bonferroni post hoc test results have been provided with significant or approaching significant *p* values. *p ≤ 0.10, **p value ≤ 0.05.

When compared by estimated sex, male and female trends echoed the results of the combined samples, displaying the highest frailty values in the Late Middle Ages, decreasing into the Contemporary Era. However, when male and female frailty values were compared overall and by period, results varied based on methodological approach (Table 5). For example, general linear models showed no significant differences by estimated sex for GHHP values, approaching statistically significant differences by sex for SFI values, and statistically significant differences by sex for BIF values (higher BIF for males than females). Furthermore, when period, estimated sex, and age group were assessed as correlative variables, none of the methods yielded similar results (Table 6). According to GLM results, significant differences using GHHP values were largely reflective of the period; significant differences using SFI reflected age and combined age‐period variables; and significant differences using BIF were observed between estimated sexes.

**TABLE 6:** Summary of general linear model (GLM) results, showing interactions between independent (period, estimated sex, and age group) and dependent (GHHP, SFI, and BIF) variables.

Variables	GLM p
GHHP ~ period	< 0.001**
GHHP ~ period + sex	0.125
GHHP ~ period + age group	0.059*
GHHP ~ sex	0.752
GHHP ~ sex + age group	0.451
GHHP ~ age group	0.105
GHHP ~ period + sex + age group	0.210
SFI ~ period	0.074*
SFI ~ period + sex	0.304
SFI ~ period + age group	0.035**
SFI ~ sex	0.087*
SFI ~ sex + age group	0.204
SFI ~ age group	0.011**
SFI ~ period + sex + age group	0.681
BIF ~ period	0.061*
BIF ~ period + sex	0.182
BIF ~ period + age group	0.366
BIF ~ sex	0.024**
BIF ~ sex + age group	0.978
BIF ~ age group	0.954
BIF ~ period + sex + age group	0.832

*Note:* *p ≤ 0.10, **p value ≤ 0.05.

## 4 Discussion

### 4.1 Comparability of Indices

Spearman's correlations demonstrated significant and positive correlations between indices. While these findings confirm that GHHP, SFI, and BIF are broadly displaying similar results, the correlation coefficients (rho) convey the limitations of their comparability. Only GHHP and SFI were highly correlated (rho = 0.531), which is interesting, as the biomarkers used by BIF were based on SFI biomarkers. Exploring this aspect with a larger sample and less restrictive preservation conditions could yield meaningful insights. The comparability of indices is further reflected in the differences observed when comparing indices by period, estimated sex, and age groups. First, results from ANOVAs vary by method. Although significant differences were observed overall by period for GHHP and BIF values (approaching significance for SFI), the differences were observed between different periods. Furthermore, significant differences by age were only noted in SFI values, and significant differences between estimated males and females were only observed in BIF values. Next, general linear models explored possible interactions between period, estimated sex, and age in relation to frailty indices. As with ANOVAs, these GLM results varied by GHHP, SFI, and BIF. There were significant and approaching significant differences observed between periods for all methodological approaches, but most of the other statistically significant or approaching significant differences were inconsistent across approaches. Measuring frailty through GHHP only yielded differences by period and age groups; measuring frailty through SFI only showed differences by period, age group, and period and age group; and lastly, measuring frailty through BIF resulted in differences by period and sex. Consequently, how a researcher interprets frailty in Milan would vary based on the methodological approach they employed.

### 4.2 Diachronic View of Frailty in Milan

This discussion of frailty in Milan is limited to findings that were relatively similar between methodological approaches. The frailty indices revealed a general trend of increasing frailty from the Roman period until the Late Middle Ages, during which the highest values of the distribution were observed. Subsequently, index values decrease into the Modern Era followed by a slight increase into the Contemporary period. Although the indices followed a similar pattern in frailty/health, statistical comparisons of these values by period, estimated sex, and estimated age yielded different findings, whether by ANOVA or GLM, based on approach. While the discussion below reflects the general trends by period—there were significant or approaching significant differences in frailty data between periods for all approaches—these results should be taken cautiously as GHHP, SFI, and BIF values varied by sex and age.

Increases in frequency of various stress markers were found from the Roman to Early Middle Ages (Table 7), namely, linear enamel hypoplasia, periodontal disease, and dental health, osteoarthrosis, trauma, rickets/osteomalacia, and porotic hyperostosis. This is consistent with historical sources that describe a worsening of living conditions with the transformation of the Western Roman Empire, deeming the early Middle Ages city overcrowded, with poorly ventilated spaces and poor hygiene following the decline of Roman aqueduct systems. Contact with contaminated water from rivers and infected wells posed a constant risk of infectious diseases, particularly dangerous for a social class already vulnerable due to food scarcity (Waaler 2002; Roberts and Manchester 2010). In the Late Middle Ages, this increase in stress markers continued (Table 7) with higher frequencies of nonspecific periostitis/osteomyelitis, vertebral diseases, degenerative joint diseases, osteoarthrosis, trauma, and cribrotic lesions (i.e., cribra orbitalia and porotic hyperostosis). In particular, this period reported the highest frequencies of enamel hypoplasia, periodontal disorders, non‐specific periostitis/osteomyelitis, and porotic hyperostosis. All three methods showed the highest mean frailty for this period, suggesting increased risk of morbidity and mortality.

**TABLE 7:** Observed stress markers, expressed as percentages, for the period and for the subsample based on methodology.

Stress marker	Roman era			Early middle ages			Late middle ages			Modern era			Contemporary era			Total		
	GHHP	SFI	BIF	GHHP	SFI	BIF	GHHP	SFI	BIF	GHHP	SFI	BIF	GHHP	SFI	BIF	GHHP	SFI	BIF
Short stature	26.1	—	22.4	32.1	—	25.0	30.6	—	20.8	15.4	—	16.7	26.3	—	24.0	27.7	—	21.8
Low body mass	—	—	18.4	—	—	18.8	—	—	14.6	—	—	18.8	—	—	22.0	—	—	18.5
Enamel hypoplasia	52.2	50.0	36.7	71.4	69.7	47.9	58.3	61.5	52.1	15.4	53.3	14.6	0.0	0.0	0.0	46.2	50.4	30.0
Peridontal disease	—	15.6	12.2	—	30.3	14.6	—	28.2	25.0	—	20.0	8.3	—	13.6	6.0	—	22.7	13.2
Dental health (abscesses)	8.7	—	—	21.4	—	—	30.6	—	—	15.4	—	—	10.5	—	—	19.3	—	—
Anemia	8.7	—	—	7.1	—	—	41.7	—	—	46.2	—	—	15.8	—	—	23.5	—	—
Cribra orbitalia	—	—	16.3	—	—	4.2	—	—	22.9	—	—	33.3	—	—	10.0	—	—	17.3
Porotic hyperostosis	—	—	0.0	—	—	4.2	—	—	12.5	—	—	10.4	—	—	0.0	—	—	5.3
Periosteal reactions	60.9	18.8	55.1	53.6	18.2	41.7	63.9	35.9	60.4	7.7	20.0	45.8	26.3	27.3	24.0	48.7	24.8	45.3
Degenerative joint disease	69.6	—	—	50.0	—	—	75.0	—		69.2	—	—	94.7	—	—	70.6	—	—
Intervertebral disease	—	46.9	—	—	42.4	—	—	59.0		—	60.0	—	—	77.3	—	—	55.3	—
Osteoarthrosis	—	21.9	18.4	—	30.3	35.4	—	46.2	58.3	—	40.0	60.4	—	77.3	74.0	—	41.1	49.4
Vertebral disease	—	—	53.1	—	—	47.9	—	—	62.5	—	—	70.8	—	—	88.0	—	—	64.6
Trauma	17.4	40.6	32.7	21.4	42.4	43.8	50.0	59.0	52.1	23.1	33.3	41.7	36.8	50.0	62.0	31.9	46.8	46.5
Rickets/osteomalacia	—	—	0.0	—	—	6.3	—	—	2.1	—	—	4.2	—	—	4.0	—	—	3.3

The Modern Era sample showed a decrease in mean frailty (Table 4), although it should be noted that fewer individuals met the criteria for GHHP and SFI methods. In particular, stress markers related to infectious events, periodontal diseases, and enamel defects decreased from the Late Middle Ages. However, as living conditions improved in the Modern Era, these results may be the result of hidden heterogeneous frailty or selective mortality (Wood et al. 1992).

In the Contemporary Era, frailty indices were slightly higher than those calculated for the Modern Era sample, although this is only significant for BIF measures. A true depiction of this era's frailty, however, cannot be derived solely from observed trends. In this era, a substantial rise in average lifespan was noted, as 72% of the estimated age‐at‐death intervals fell above 60 years. Biomechanical stress (e.g., DJD, vertebral diseases, and trauma) was consistently among the highest in this historical interval (Table 7), and the accumulation of biomarkers related to geriatric conditions may have resulted in higher frailty scores. The Health Index and the SFI were less applicable, with less than 50% of individuals meeting criteria for observable conditions (unlike the method by Zedda, which was applicable to all individuals). As already mentioned for the Modern Era, this limitation leads us to question sample representativeness for GHHP and SFI results. In particular, a major methodological limitation was the frequent inability to score dental stress markers due to antemortem tooth loss. Edentulism is a typical consequence of aging, but other factors include hygiene, health status, socioeconomic status, or even taphonomy. Complete antemortem tooth loss is known to impact health (Emami et al. 2013), and should be considered in frailty assessments rather than being seen as a hindrance. Moreover, this limitation may have led to misrepresentations in frailty and health interpretations, as numerous stress markers could not be evaluated.

When estimated sex was considered, male and female patterns generally followed those of the overall sample, with the Late Middle Ages exhibiting the highest frailty values. While all three indices displayed similar increases and decreases in frailty for males, this was only the case for females with SFI and BIF values, as GHHP values showed the Early Middle Ages sample to be the frailest. According to all three tested methods, the only significant difference in frailty between sexes was observed in the Roman sample. Specifically, females exhibited significantly lower frailty scores than males for GHHP, SFI, and BIF. This difference might be related to the fact that male individuals were traditionally more involved in physical labor and were therefore more exposed to the accumulation of stress markers related to strenuous tasks and a higher risk of accidents (Rodella et al. 2022). In previous studies, Roman period females in Milan showed younger ages at death with respect to their male counterparts, and this increased female mortality was partly attributed to the risks related to pregnancy and childbirth (Rodella et al. 2022). This earlier mortality in Roman females might explain the accumulation of fewer stress markers and higher frailty values compared to males. While further dissection of male–female frailty differences is important, it is currently beyond the scope of this methodological paper and covered extensively in another publication (Biehler‐Gomez et al. 2024).

### 4.3 The Health Index: An Innovative but Imperfect Index

Frailty indices represent a relatively novel practice aimed at studying a fundamental theme of bioarchaeological research, that of interpreting “health” in past populations. The possibilities these methods offer, however, are much greater, as they represent a tool for detecting inequalities in the past and present through diachronic analyses. Additionally, indices of frailty facilitate comparisons across populations and thus encourage knowledge and data sharing between researchers.

To the best of the authors' knowledge, the technique proposed by Steckel and colleagues has seen very few applications (Hubbe et al. 2018). Therefore, the creation of the SFI by Marklein et al. (2016) reignited the study of skeletal frailty and, in conjunction with the BIF (Zedda et al. 2021), simplified its practice. Recent applications demonstrate how valuable these methodologies can be as they challenge previously established hypotheses about health trends (Gaddis 2018; Kyle et al. 2018; Dafoe 2020; Tuggle et al. 2021; Frazier 2022; Marklein and Crews 2022; Paliulytė 2024).

One of the elements that limited the application of the Steckel and Rose method was data saturation. Indeed, the method is lengthy and, at times, cumbersome: it requires numerous parameters (i.e., 24 elements) for which scoring is required according to the authors' specifications, followed by a secondary conversion into final scores. While a methodological approach with detailed data can support thorough investigation and high‐quality data analysis, the approach proposed by the authors leads to loss of information. A notable example is the trauma assessment system: for each individual, observation and scoring of seven skeletal regions are required, each with its own evaluation criteria and methodological indications. The stress marker scoring corresponds to the lowest observed value, yet the time invested in diagnosing and describing traumatic phenomena—that ultimately are not represented—is effectively useless. Furthermore, several skeletal regions—such as the spine, pectoral and pelvic girdles, rib cage, and feet—are entirely neglected in the assessment pool. Consequently, various trauma cases diagnosed during the anthropological analysis were excluded from the frailty calculation. Similar issues were also identified regarding the “Degenerative Joint Disease” category.

Moreover, the criteria for evaluation provided by the authors proved to be vague, often recurring to phrasing such as “[ …] only extreme deterioration is recorded […],” “[…] with acceptable alignment” or “extensive osteophyte formation […]” (Steckel, Sciulli, et al. 2002, 90, 91). While these semantic choices grant future specialists the freedom to expand investigation criteria to more modern and reliable practices, relying on vague language inevitably leads to confusion. During analysis, this necessitated the retrieval and sometimes repetition of skeletal material analyses to recalibrate certain assigned scores. Rigorous terminological and methodological precision is crucial to ensure experimental repeatability.

Because of these ambiguous instructions, mathematical inconsistencies were found in the “completeness” component of the “dental health” marker calculations. Calculations often resulted in anomalous figures, disproportionately high or even negative, and were thus not counted in the final index. The scoring for this stress marker is defined as follows: “Completeness is defined by one minus the ratio of the sum of premortem loss and cavities to the sum of teeth and premortem loss. The sum of teeth and premortem loss must be eight or more; otherwise, data in this category are deemed incomplete and are not used” (Steckel, Rose, et al. 2002, 147). There is ambiguity regarding what exactly is meant by “cavities”; in dental terminology, this term also refers to the occurrence of dental caries.

Further major issues were also found concerning the weighting of the evaluations: “All attributes of health are weighted equally in the index. While it may be difficult to justify this assumption, given the present state of knowledge it is also difficult to justify any particular set of alternative weights” (Steckel, Sciulli, et al. 2002, 69). Despite the authors' neutral stance on the weight of stress markers, the same cannot be said for the respective scoring parameters because of the evaluative system in “categories” that the method uses. Certain anatomical regions are much more susceptible to extreme scoring assignments of 0 or 100 compared to others. For instance, a scoring of 0 for “shoulders and elbows” in the “Degenerative Joint Disease” category would require observing “immobilization of the joint attributable to degenerative processes,” a far more severe and rare phenomenon. In contrast, to assign the same score—which the authors define as “the worst possible health condition for the stress marker”—to hands, merely observing “degenerative phenomena” suffices. This discrepancy results in an undeniable overestimation of frailty.

Due to methodological limitations recognized by the authors themselves, the index was also referred to as “Mark I.” Although measurement techniques were provided in the publication, they were considered provisional tools, and the implementation of updated methodologies was encouraged (Steckel, Sciulli, et al. 2002). Similarly, while the equal weighting of biomarkers may be difficult to justify, assigning different weights was unjustifiable at the time of the index proposal, providing a basis for incorporating updated methodologies.

Finally, the last critique of the Health Index method includes its use of statistically calibrated synthetic reference populations. The advantage of using such criteria undoubtedly lies in the ability to compare values directly with contemporary living populations. However, as discussed in the Osteological Paradox, Wood et al. (1992) illustrate how “non‐demographic stationarity,” “selective mortality,” and “hidden heterogeneity” render this practice less meaningful. Accordingly, subsequent attempts to calculate frailty have abandoned this approach in favor of studying directly comparable elements.

### 4.4 SFI and BIF: Improvements and New Challenges

In an effort to address the limited applicability of the original SFI (Marklein et al. 2016), the revision of the method explored the statistical validity of alternative combinations of biomarkers. Among these, the 6‐marker stress index emerged as the most promising, as it presented statistical robustness comparable to the original. However, as the revised index was developed by sequentially eliminating biomarkers based on frequency and aimed at maximizing validation within the Medieval London study sample, this 6‐biomarker SFI was therefore constructed expressly to test frailty in Medieval London and thus may not be appropriate for other populations. As stated (Marklein and Crews 2017), two of the markers that significantly constrained their sample size were “maximum femoral length” and “femoral head diameter,” both traits abundantly recorded in the current Milanese skeletal population, with 208 and 185 valid measurements respectively. This underlines the challenge of building frailty models based on population‐specific stress markers, specifically, the poor reproducibility of findings beyond their contextual relevance. Additionally, the six‐biomarker SFI was overly represented by mechanical stress markers—joint degeneration, trauma, and degenerative vertebral diseases (three out of six)—thus hindering a fair depiction of health status. Moving toward a non‐population specific index, equipped to withstand the analysis of partial skeletons, and “ready‐for‐use” system would be preferable.

Zedda et al. (2021) suggested that the BIF “surpass[es] the limitations of previously proposed [indices]” (Zedda et al. 2021). Our study demonstrates that, indeed in this sample, the BIF had a higher applicability rate than the Health Index and SFI, enabling the evaluation of 97% of individuals, even with significantly incomplete skeletons. As long as at least three stress biomarkers were scored, the index could be calculated even for incomplete remains, thereby allowing comparisons with complete and more represented individuals. However, this raises questions about the actual reliability of comparisons permitted by this method, as, realistically, partial and incomplete individuals are not directly comparable. Another point of criticism of the BIF is the weighting system for stress markers. While this allows for tracking the severity and remodeling of lesions (reasonably cited as an improvement by the authors), behind this criterion lies the hypothesis that individuals who died prematurely exhibit biomarkers that contribute more significantly to individual frailty. Weights are thus assigned through logistic models that are population‐specific, a fact that may add complications to the comparisons between unrelated populations.

Ultimately, one of the major challenges of health and frailty indices lies in their applicability. In this study, selection criteria explicitly revolved around preservation and a reliable sex estimation. As far as archaeological context goes, such conditions can be rare; in fact, good preservation tends to diminish as we go further back in time (Reitsema and McIlvaine 2014; Biehler‐Gomez et al. 2022). The situation therefore presented here approximates an ideal scenario, yet half of the selected individuals were excluded. Although the SFI adapted its requirements to improve applicability, only 56% of the original 250‐individual sample was considered. Thus, two (Health Index and SFI) of the three tested methods could assess only half or less than half of the original sample. These methods were notably conservative, requiring observations on all stress markers, which inherently depended on skeletal preservation and completeness. It seems improbable that half or fewer of the selected individuals could reliably represent each of the five historical periods. While the obvious solution might be to consider larger skeletal populations, the present study showcases the limitations of cumulative indexical approaches to frailty. In contrast, the BIF proved to be the most representative in terms of sample size: its flexibility, requiring a minimum of three evaluations, enabled index calculation for over 97% of the sample. However, this approach introduces a significant challenge: poorly preserved individuals, with indices calculated on fewer markers, are compared with individuals in excellent condition, raising grave interpretational concerns, especially when considering the osteological paradox.

### 4.5 Future of Health Indices

Selection, scoring, and analysis of biomarkers inherently pose a dilemma for bioarchaeologists, as they may be possible indicators of frailty, resilience, or population variation, depending on thecontext.

For selection considerations, methods tend to focus on detecting lesions of infection in the tibiae, disregarding other manifestations if these bones are not observable as it is considered a common site for detecting non‐specific periostitis. However, this should not be a sufficient reason to limit observations. Although the Health Index acknowledged the possibility of periostitis outside the tibiae, it unjustifiably assigned a more significant score to “tibial reactions” The BIF took the issue into consideration, but the method stagnated in requiring at least one tibia for validation. Only the SFI method partly removed this limitation. Periostitis is the most generic response of the bone tissue (Cramer 2018), a stress marker that can occur in virtually any skeletal site. Disregarding this may result in underrepresentation of the condition, hinder paleoepidemiological considerations, and lead to less realistic frailty assessments.

As it relates to the scoring of biomarkers, the methods placed considerable emphasis on observing fractures and signs of their healing. However, “fractures” represent only a fraction of traumatic injuries, whose manifestations are known to include traumatic calcifications, avascular necrosis, and traumatic soft tissue injuries responsible for clinical contexts of unwellness, such as osteochondritis dissecans and myositis ossificans (Clanton and DeLee 1982; Rodríguez‐Martín 2006; Walczak et al. 2015; Bacci et al. 2019; Biehler‐Gomez and Cattaneo 2021), which are also important to understanding frailty. Although not addressed in the SFI and BIF, Marklein et al. (2016) and Zedda et al. (2021) mention the incorporation of other such biomarkers into future indices. Given the general issue of trauma underestimation that plagues anthropological assessment (Domett and Tayles 2006), it is therefore recommended to broaden its definition. It would also be advisable to explore trauma recidivism (Judd 2002; Redfern et al. 2017; Biehler‐Gomez, Moro, et al. 2023), especially as the link between multiple traumas and victims' lifestyles in the context of chronic illness (Sims et al. 1989) could prove to be a stress factor equal to others more traditionally taken into consideration.

Although the spine is a region mentioned in all three methods, numerous associated conditions are not reported. Schmorl's nodes, for example, are detected in up to 75% of the population (Hilton et al. 1976; Kyere et al. 2012; Sonne‐Holm et al. 2013; Jagannathan et al. 2016) and, even if not considered stress markers per se, should at least be considered in the criteria for IVD or other degenerative joint conditions. These lesions resulting from the herniation of the intervertebral disc nucleus have been excluded from the evaluation criteria of all methods, which instead focus on osteophytes, osteoarthritis, and ankylosis.

In both the Health Index and BIF, frailty assessment considered cribrotic lesions. However, attention was solely focused on cribra orbitalia and porotic hyperostosis, despite literature also noting the existence and relevance of cribra humeralis and femoralis (Djuric et al. 2008; Göhring 2021; Schats 2021). In fact, in the present study, while the cribra orbitalia and porotic hyperostosis were observable in 52 cases, the inclusion of other cribriotic variants increased the count to 60, demonstrating that a broader definition may lead to an increase in the applicability of the index.

Additionally, proposals may be made for the addition of other stress markers. For instance, Harris lines appear as sclerotized bands with a linear pattern, immersed in the medullary cavity of long bones, observable through X‐ray instrumentation (Harris 1931). Whether they are considered “growth arrest lines” or “recovery lines” (Park and Richter 1953; Alfonso et al. 2005), Harris lines have been seen as a biological marker of physiological stress reflecting malnutrition and unhealthy growth environments (Inokuchi et al. 2000; Kulus and Dąbrowski 2019). However, this marker was never included in the frailty indices. In the present study, this possibility was contemplated (but not included in the assestment), and the biomarker resulted visible in 59 of the 250 skeletons of the study sample.

Recently, a new approach for quantifying frailty and resilience in the past has been proposed by Yaussy and colleagues (2024). This method employs survival (Kaplan–Meier) and hazard (Cox proportional and Gompertz) analyses (Yaussy et al. 2024), effectively enabling researchers to build a frailty index with biomarkers that are associated with higher mortality in a specific population. While this approach considers the osteological paradox, one question that remains is the comparability of the results between different populations, as this construction of frailty indices is effectively based in “local biologies” (Lock 1993; Kontos 1999). If population‐specific frailty indices are the best means of interpreting frailty and resilience in one population, how do we compare frailty between populations with different population‐specific indices?

## 5 Conclusion

After considering these indices and the limitations therein, the question arises: is the term “frailty” still appropriate? Initially conceived as a predictive tool to quantify “vulnerability,” it has undergone considerable reevaluation over time. Indeed, the debate has been widely misunderstood and frequently addressed with insufficient consideration of the Osteological Paradox. Only recently has there been renewed interest in the topic, leading to the progressive reconceptualization of the concept and the corresponding revisiting of the original methodologies to quantify it. Frailty is now seen as an expression of adaptive plasticity.

This study, to the best of our knowledge, is the first to compare bioarchaeological frailty indices (Health Index, SFI, and BIF) directly, through correlations, and indirectly, through diachronic analyses in urban Milan. Their applicability proved to be heavily influenced by the preservation of skeletons and, at times, even by oversights in the design of the methods themselves. Moreover, direct comparison between indices was complicated by the often context‐specific approach by which stress markers are selected. This paper demonstrates how these methods should not be considered the “only” or “best” approach for quantifying “skeletal frailty.” Yet even with methodological limitations and sample representativeness, these methods provided alternative, cumulative approaches to mapping and interpreting frailty in Milan. Since the results from the three methods agree on the general trend, they could collectively become a valuable tool for bioarchaeologists to gain deeper insight into the environmental‐health dynamics within archaeological settings. However, when assessing cumulative skeletal frailty in the past, researchers should select their methodological approach intentionally, as all indices do not yield the same results.

## Author Contributions

**D. Petrosino:** conceptualization (supporting), formal analysis (lead), investigation (equal), methodology (equal), writing – original draft (equal), writing – review and editing (equal). **L. Biehler‐Gomez:** conceptualization (lead), formal analysis (supporting), investigation (equal), methodology (equal), writing – original draft (equal), writing – review and editing (equal). **K.E. Marklein:** formal analysis (equal), investigation (equal), methodology (equal), writing – review and editing (equal). **M. Mondellini:** formal analysis (equal). **C. Moro:** formal analysis (equal). **M. Mattia:** data curation (equal). **A.M. Fedeli:** resources (equal). **C. Cattaneo:** supervision (equal).

## Conflicts of Interest

The authors declare no conflicts of interest.

## Supporting information


**Data S1.** Supporting Information.

## Data Availability

All data generated and analyzed during this study are included in this published article.

## Endnotes

^1^

https://www.ilgiorno.it/milano/cronaca/differenze‐di‐genere‐e‐longevita‐tra‐rischi‐diritti‐e‐lavori‐usuranti‐cosi‐e‐cambiata‐la‐vita‐delle‐donne‐a89f8a4a.

^2^

https://www.cronachediscienza.it/2024/10/16/un‐nuovo‐studio‐riscrive‐la‐storia‐della‐mortalita‐e‐della‐sopravvivenza‐femminile‐a‐milano‐negli‐ultimi‐2mila‐anni/.
