# Supplementary material for: “We Are the Sons of Our Own Deeds”: Comparing Skeletal Health and Frailty Indices in Deceased Individuals Across 2000 Years of Milanese History
Source: Am J Biol Anthropol. 2025 Mar 19;186(3):e70025. doi: 10.1002/ajpa.70025 (PMC11923401; doi:10.1002/ajpa.70025)
Supplement: Supplementary file 1 — Data S1. Supporting Information. [file AJPA-186-e70025-s001.docx]

**Supplementary Material**

These materials contain the archival data for all 250 individuals in the study sample, organized by historical period, along with the results of the calculations for the three indices. Cases where the calculation was not possible are marked with an "-".

| Period | Site | Individual ID | Biological Sex | Age-at-death | Steckel & Rose - “Health index” (2002) | Marklein et al. - SFI  (2016 – 2017) | Zedda et al. - BIF  (2021) |
| --- | --- | --- | --- | --- | --- | --- | --- |
| Roman Era | Cattolica | 265 | M | 31-45 | 32.14 | 33.33 | 50.00 |
| Roman Era | Cattolica | 415 | F | 31-45 | 35.71 | 16.67 | 33.33 |
| Roman Era | Cattolica | 832 | F | 31-45 | 12.00 | 16.67 | 21.05 |
| Roman Era | Cattolica | 868 | F | 21-30 | - | - | 33.33 |
| Roman Era | Cattolica | 1513 | F | 16-20 | - | 0.00 | 0.00 |
| Roman Era | Cattolica | 1709 | M | 46-60 | 38.29 | 66.67 | 60.00 |
| Roman Era | Cattolica | 1757 | F | 16-20 | - | - | 33.33 |
| Roman Era | Cattolica | 1878 | M | 31-45 | - | - | 50.00 |
| Roman Era | Cattolica | 1962 | M | 46-60 | - | - | 57.14 |
| Roman Era | Cattolica | 1970 | F | 31-45 | 37.00 | 33.33 | 15.38 |
| Roman Era | Cattolica | 2061 | M | 31-45 | 19.14 | 33.33 | 35.00 |
| Roman Era | Cattolica | 2270 | M | 21-30 | - | 16.67 | 50.00 |
| Roman Era | Cattolica | 2464 | M | 16-20 | - | 50.00 | 57.14 |
| Roman Era | Cattolica | 2950 | M | 31-45 | - | - | 20.00 |
| Roman Era | Cattolica | 2995 | M | 61-80 | 32.14 | 66.67 | 30.00 |
| Roman Era | Cattolica | 3054 | F | 46-60 | 19.14 | 50.00 | 47.62 |
| Roman Era | Cattolica | 3062 | F | 16-20 | 14.29 | 16.67 | 11.76 |
| Roman Era | Cattolica | 3097 | M | 46-60 | - | - | 50.00 |
| Roman Era | Cattolica | 3117 | F | 21-30 | - | 33.33 | 21.43 |
| Roman Era | Cattolica | 3129 | F | 21-30 | - | 33.33 | 27.27 |
| Roman Era | Cattolica | 3138 | M | 31-45 | 38.14 | 50.00 | 45.00 |
| Roman Era | Cattolica | 3181 | M | 31-45 | 47.71 | 50.00 | 50.00 |
| Roman Era | Cattolica | 3263 | F | 31-45 | 23.86 | 50.00 | 28.57 |
| Roman Era | Cattolica | 3272 | M | 31-45 | - | - | 25.00 |
| Roman Era | Cattolica | 3278 | F | 46-60 | 14.43 | 50.00 | 20.00 |
| Roman Era | Cattolica | 3301 | M | 31-45 | - | - | 5.88 |
| Roman Era | Cattolica | 3307 | M | 46-60 | - | - | 26.67 |
| Roman Era | Cattolica | 3336 | F | 21-30 | 23.86 | 16.67 | 5.00 |
| Roman Era | Cattolica | 3350 | M | 31-45 | 28.57 | 66.67 | 55.00 |
| Roman Era | Cattolica | 3373 | F | 21-30 | - | - | - |
| Roman Era | Cattolica | 3425 | M | 31-45 | 23.86 | 33.33 | 19.05 |
| Roman Era | Cattolica | 3465 | F | 21-30 | - | - | 18.75 |
| Roman Era | Cattolica | 3644 | F | 21-30 | 28.57 | 33.33 | 30.00 |
| Roman Era | Cattolica | 3716 | F | 16-20 | - | - | 14.29 |
| Roman Era | Cattolica | 3959 | F | 31-45 | - | 0.00 | 0.00 |
| Roman Era | Cattolica | 4027 | M | 46-60 | 9.57 | 50.00 | 22.22 |
| Roman Era | Cattolica | 4115 | F | 46-60 | - | - | 12.50 |
| Roman Era | Cattolica | 4191 | M | 46-60 | 35.71 | 50.00 | 30.00 |
| Roman Era | Cattolica | 5290 | F | 31-45 | - | - | 23.53 |
| Roman Era | Cattolica | 5890 | M | 46-60 | 20.29 | 50.00 | 25.00 |
| Roman Era | Cattolica | 7375 | F | 31-45 | - | - | 0.00 |
| Roman Era | Cattolica | 275 A-1 | M | 31-45 | - | 0.00 | 16.67 |
| Roman Era | Cattolica | 275 A-2 | M | 21-30 | - | 16.67 | 40.00 |
| Roman Era | Cattolica | 283 A | F | 21-30 | - | 16.67 | 0.00 |
| Roman Era | Cattolica | 3041-3141 | F | 31-45 | 0.00 | 0.00 | 15.79 |
| Roman Era | Cattolica | 3471 A | M | 31-45 | 38.14 | 33.33 | 23.81 |
| Roman Era | Cattolica | 3471 B | F | 21-30 | 14.29 | 0.00 | 13.33 |
| Roman Era | Cattolica | 414 A | F | 21-30 | - | - | 43.75 |
| Roman Era | Cattolica | 437 - 2989 | M | 31-45 | - | - | 72.73 |
| Roman Era | Cattolica | 456 - 3098 | M | 31-45 | - | - | 0.00 |
| Early Middle Ages | S. Ambrogio | 123 | F | 21-30 | 50.00 | 33.33 | 40.00 |
| Early Middle Ages | S. Ambrogio | 134 | M | 46-60 | - | - | 54.55 |
| Early Middle Ages | S. Ambrogio | 135 | F | 46-60 | - | 33.33 | 23.08 |
| Early Middle Ages | S. Ambrogio | 173 | F | 46-60 | 47.71 | 66.67 | 71.43 |
| Early Middle Ages | S. Ambrogio | 210 | F | 31-45 | 10.71 | 33.33 | 55.00 |
| Early Middle Ages | S. Ambrogio | 212 | F | 31-45 | - | 16.67 | 44.44 |
| Early Middle Ages | S. Ambrogio | 223 | M | 46-60 | 20.29 | 66.67 | 33.33 |
| Early Middle Ages | S. Ambrogio | 230 | F | 21-30 | - | - | 38.10 |
| Early Middle Ages | S. Ambrogio | 234 | M | 31-45 | 0.00 | 0.00 | 66.67 |
| Early Middle Ages | S. Ambrogio | 243 | M | 31-45 | - | - | 19.05 |
| Early Middle Ages | S. Ambrogio | 258 | M | 31-45 | - | - | 55.56 |
| Early Middle Ages | S. Ambrogio | 261 | M | 21-30 | 14.29 | 16.67 | 62.50 |
| Early Middle Ages | S. Ambrogio | 267 | F | 46-60 | 35.71 | 66.67 | 19.05 |
| Early Middle Ages | S. Ambrogio | 268 | M | 21-30 | - | 66.67 | 19.05 |
| Early Middle Ages | S. Ambrogio | 275 | M | 21-30 | 42.86 | 16.67 | 42.11 |
| Early Middle Ages | S. Ambrogio | 286 | M | 16-20 | - |  | 9.52 |
| Early Middle Ages | S. Ambrogio | 288 | F | 46-60 | 47.33 | 50.00 | - |
| Early Middle Ages | S. Ambrogio | 290 | F | 21-30 | 40.57 | 50.00 | 23.81 |
| Early Middle Ages | S. Ambrogio | 292bis | F | 16-20 | 28.57 | 33.33 | 33.33 |
| Early Middle Ages | S. Ambrogio | 294 | F | 16-20 | - | - | 0.00 |
| Early Middle Ages | S. Ambrogio | 299 | F | 31-45 | 0.00 | 0,00 | 0.00 |
| Early Middle Ages | S. Ambrogio | 301 | M | 16-20 | 57.14 | 66.67 | 78.95 |
| Early Middle Ages | S. Ambrogio | 305 | M | 46-60 | - | - | 27.27 |
| Early Middle Ages | S. Ambrogio | 136-B | F | 31-45 | - | - | 54.55 |
| Early Middle Ages | S. Ambrogio | 141-A | M | 16-20 | - | - | 0.00 |
| Early Middle Ages | S. Ambrogio | 141-B | M | 46-60 | - | - | 50.00 |
| Early Middle Ages | S. Ambrogio | 187 - A | M | 46-60 | 42.86 | 83.33 | 40.00 |
| Early Middle Ages | S. Ambrogio | 187 - B | M | 46-60 | - | 0.00 | 6.67 |
| Early Middle Ages | S. Ambrogio | 192-B | F | 31-45 | - | - | 27.27 |
| Early Middle Ages | S. Ambrogio | 229-A | M | 31-45 | - | - | 30.00 |
| Early Middle Ages | S. Ambrogio | 229-B | F | 31-45 | - | - | 25.00 |
| Early Middle Ages | S. Ambrogio | 245 US 901 | M | 46-60 | 56.00 | 66.67 | 73.33 |
| Early Middle Ages | S. Ambrogio | 245 US 952 | M | 46-60 | 41.71 | 66.67 | 25.00 |
| Early Middle Ages | S. Ambrogio | 296-A | F | 31-45 | 33.43 | 66.67 | 30.00 |
| Early Middle Ages | S. Ambrogio | 296-B | M | 21-30 | - | 16.67 | 40.00 |
| Early Middle Ages | S. Ambrogio | US 1116-A | F | 21-30 | 31.00 | 16.67 | 19.05 |
| Early Middle Ages | S. Ambrogio | US 1116-B | F | 46-60 | 28.71 | 66.67 | 0.00 |
| Early Middle Ages | San Vittore | 23 - IND A1 | M | 21-30 | 24.00 | 66.67 | 33.33 |
| Early Middle Ages | San Vittore | 23 - IND B1 | F | 21-30 | 14.29 | 16.67 | 22.22 |
| Early Middle Ages | San Vittore | 24 | M | 46-60 |  | 33.33 | 38.10 |
| Early Middle Ages | San Vittore | 28 - IND A1 | M | 21-30 | 14.29 | 16.67 | 0.00 |
| Early Middle Ages | San Vittore | 28 - IND B1 | F | 31-45 | 28.57 | 0.00 | 54.55 |
| Early Middle Ages | San Vittore | 29 - IND A1 | M | 21-30 | 0.00 | 16.67 | 30.77 |
| Early Middle Ages | San Vittore | 31 - IND A1 | F | 31-45 | - | - | 15.00 |
| Early Middle Ages | San Vittore | 31 - IND B1 | M | 31-45 | 23.86 | 16.67 | 40.00 |
| Early Middle Ages | San Vittore | 35 - IND A | M | 31-45 | 4.17 | - | - |
| Early Middle Ages | San Vittore | 35 - IND C | F | 46-60 | 27.83 | - | 33.33 |
| Early Middle Ages | San Vittore | 36 - IND A | F | 21-30 | - | - | 50.00 |
| Early Middle Ages | San Vittore | 40 - IND A1 | F | 46-60 | - | 50.00 | 36.84 |
| Early Middle Ages | San Vittore | 69 - IND A | F | 46-60 | 33.43 | 66.67 | 50.00 |
| Late Middle Ages | S. Ambrogio | 4 | M | 21-30 | 52.43 | 66.67 | 52.94 |
| Late Middle Ages | S. Ambrogio | 6 | F | 21-30 | 0.00 | 16.67 | 10.00 |
| Late Middle Ages | S. Ambrogio | 7 | F | 46-60 | 38.14 | 83.33 | 29.41 |
| Late Middle Ages | S. Ambrogio | 8 | F | 16-20 | - | - | 50.00 |
| Late Middle Ages | S. Ambrogio | 15 | M | 21-30 | 26.29 | 50.00 | 28.57 |
| Late Middle Ages | S. Ambrogio | 20 | M | 46-60 | 12.00 | 50.00 | 25.00 |
| Late Middle Ages | S. Ambrogio | 26 | F | 16-20 | 28.71 | 33.33 | 30.00 |
| Late Middle Ages | S. Ambrogio | 28 | M | 31-45 | 46.43 | 100.00 | 70.59 |
| Late Middle Ages | S. Ambrogio | 34 | M | 31-45 | 25.00 | 16.67 | 43.75 |
| Late Middle Ages | S. Ambrogio | 39 | M | 46-60 | - | - | 80.00 |
| Late Middle Ages | S. Ambrogio | 40 | F | 46-60 | 26.29 | 50.00 | 23.81 |
| Late Middle Ages | S. Ambrogio | 46 | F | 31-45 | 44.50 | 33.33 | 37.50 |
| Late Middle Ages | S. Ambrogio | 49 | M | 21-30 | 28.57 | 16.67 | 11.11 |
| Late Middle Ages | S. Ambrogio | 64 | M | 16-20 | 32.14 | 50.00 | 61.11 |
| Late Middle Ages | S. Ambrogio | 68 | F | 16-20 | 35.71 | 33.33 | 50.00 |
| Late Middle Ages | S. Ambrogio | 71 | M | 46-60 | 32.14 | 50.00 | 50.00 |
| Late Middle Ages | S. Ambrogio | 77 | M | 16-20 | 47.71 | 33.33 | 58.33 |
| Late Middle Ages | S. Ambrogio | 78 | F | 31-45 | 20.43 | 66.67 | 47.62 |
| Late Middle Ages | S. Ambrogio | 81 | F | 46-60 | 16.71 | 16.67 | 12.50 |
| Late Middle Ages | S. Ambrogio | 87 | M | 21-30 | 32.14 | 16.67 | 25.00 |
| Late Middle Ages | S. Ambrogio | 89 | F | 31-45 | 48.86 | 83.33 | 45.00 |
| Late Middle Ages | S. Ambrogio | 94 | M | 31-45 | 51.29 | 66.67 | 64.71 |
| Late Middle Ages | S. Ambrogio | 98 | M | 21-30 | 20.29 | 33.33 | 33.33 |
| Late Middle Ages | S. Ambrogio | 103 | F | 21-30 | 21.43 | 50.00 | 57.14 |
| Late Middle Ages | S. Ambrogio | 105 | M | 46-60 | 34.57 | 50.00 | 42.86 |
| Late Middle Ages | S. Ambrogio | 132 | M | 21-30 | 33.43 | 33.33 | 38.10 |
| Late Middle Ages | S. Ambrogio | 134 | M | 46-60 | - | - | 46.15 |
| Late Middle Ages | S. Ambrogio | 135 | F | 21-30 | - | 50.00 | 72.73 |
| Late Middle Ages | S. Ambrogio | 137 | F | 46-60 | - | - | 29.41 |
| Late Middle Ages | S. Ambrogio | 138 | M | 46-60 | 59.57 | 83.33 | 45.00 |
| Late Middle Ages | S. Ambrogio | 139 | F | 46-60 | - | - | 31.25 |
| Late Middle Ages | S. Ambrogio | 144 | F | 46-60 | - | - | 42.86 |
| Late Middle Ages | S. Ambrogio | 156 | F | 31-45 | 23.86 | 50.00 | 23.53 |
| Late Middle Ages | S. Ambrogio | 163 | M | 21-30 | 36.17 | 83.33 | 52.94 |
| Late Middle Ages | S. Ambrogio | 165 | M | 31-45 | - | 33.33 | 30.77 |
| Late Middle Ages | S. Ambrogio | 104 US 336 | F | 31-45 | 27.43 | 33.33 | 23.81 |
| Late Middle Ages | S. Ambrogio | 14-A | F | 46-60 | 21.43 | 33.33 | 15.79 |
| Late Middle Ages | S. Ambrogio | 14-B | F | 21-30 | - | - | - |
| Late Middle Ages | S. Ambrogio | 142-1 | F | 46-60 | 16.67 | 16.67 | 33.33 |
| Late Middle Ages | S. Ambrogio | 142-2 | M | 46-60 | - | - | - |
| Late Middle Ages | S. Ambrogio | 160-A | F | 46-60 | 32.14 | 83.33 | 50.00 |
| Late Middle Ages | S. Ambrogio | 160-B | F | 16-20 | 54.17 | 66.67 | 64.71 |
| Late Middle Ages | S. Ambrogio | 164-1 | M | 31-45 | 33.43 | 66.67 | 23.53 |
| Late Middle Ages | S. Ambrogio | 164-2 | F | 31-45 | - | - | 37.50 |
| Late Middle Ages | S. Ambrogio | 19-A | F | 31-45 | 22.71 | - | 20.00 |
| Late Middle Ages | S. Ambrogio | 19-C | M | 31-45 | 33.43 | 50.00 | 28.57 |
| Late Middle Ages | S. Ambrogio | 65-A | F | 61-80 | 50.14 | 66.67 | 35.00 |
| Late Middle Ages | S. Ambrogio | 65-B | M | 31-45 | 19.14 | 50.00 | 26.67 |
| Late Middle Ages | S. Ambrogio | 83-A | M | 46-60 | - | 16.67 | 7.14 |
| Late Middle Ages | S. Ambrogio | 83-B | M | 31-45 | - | - | 0.00 |
| Modern Era | Viale Sabotino | GR 1 IND B | F | 46-60 | - | - | 42.86 |
| Modern Era | Viale Sabotino | GR 1 IND D | M | 21-30 | - | - | 57.14 |
| Modern Era | Viale Sabotino | GR 1 IND E | M | 31-45 | - | 83.33 | 73.68 |
| Modern Era | Viale Sabotino | GR 1 IND F | F | 61-80 | - | - | 22.22 |
| Modern Era | Viale Sabotino | GR 10 IND B | F | 21-30 | - | - | 9.09 |
| Modern Era | Viale Sabotino | GR 10 IND D’ | F | 46-60 | - | 50.00 | 25.00 |
| Modern Era | Viale Sabotino | GR 10 IND E | M | 31-45 | - | - | 50.00 |
| Modern Era | Viale Sabotino | GR 10 IND F | F | 46-60 | - | 33.33 | 29.41 |
| Modern Era | Viale Sabotino | GR 10 IND H | M | 46-60 | 0.00 | 0.00 | 22.22 |
| Modern Era | Viale Sabotino | GR 10 IND H’ | M | 46-60 | 31.00 | - | 38.46 |
| Modern Era | Viale Sabotino | GR 10 IND O | M | 31-45 | - |  | 54.55 |
| Modern Era | Viale Sabotino | GR 11 IND AA | M | 46-60 | - | 50.00 | 31.25 |
| Modern Era | Viale Sabotino | GR 11 IND BB | F | 46-60 | 10.71 | 33.33 | 29.41 |
| Modern Era | Viale Sabotino | GR 11 IND H-1 | M | 61-80 | 9.57 | - | 12.50 |
| Modern Era | Viale Sabotino | GR 11 IND H-2 | F | 46-60 | - | - | - |
| Modern Era | Viale Sabotino | GR 11 IND P | F | 31-45 | - | - | 42.86 |
| Modern Era | Viale Sabotino | GR 12 IND E | M | 46-60 | - | 66.67 | 40.00 |
| Modern Era | Viale Sabotino | GR 13 IND C | M | 21-30 | 4.86 | - | 6.67 |
| Modern Era | Viale Sabotino | GR 14 IND C | M | 46-60 | - | - | 50.00 |
| Modern Era | Viale Sabotino | GR 2 IND B | M | 61-80 | - | - | 31.25 |
| Modern Era | Viale Sabotino | GR 2 IND C | F | 31-45 | 21.43 | 66.67 | 47.62 |
| Modern Era | Viale Sabotino | GR 2 IND D | F | 46-60 | 9.57 | - | 38.89 |
| Modern Era | Viale Sabotino | GR 2 IND H | F | 31-45 | 9.57 | 16.67 | 4.76 |
| Modern Era | Viale Sabotino | GR 2 IND I | F | 46-60 | - | - | 35.29 |
| Modern Era | Viale Sabotino | GR 2 IND L | F | 16-20 | 21.43 | 16.67 | 0.00 |
| Modern Era | Viale Sabotino | GR 3 IND B | F | 21-30 | - | - | 6,25 |
| Modern Era | Viale Sabotino | GR 3 IND C | M | 46-60 | 38.14 | 50.00 | 33.33 |
| Modern Era | Viale Sabotino | GR 3 IND D | M | 46-60 | - | - | 41.67 |
| Modern Era | Viale Sabotino | GR 3 IND E | M | 46-60 | - | - | 29.41 |
| Modern Era | Viale Sabotino | GR 3 IND F | M | 46-60 | - | - | 38.89 |
| Modern Era | Viale Sabotino | GR 3 IND I | M | 21-30 | 15.57 | 33.33 | 50.00 |
| Modern Era | Viale Sabotino | GR 3 IND L | F | 46-60 | - | - | 0.00 |
| Modern Era | Viale Sabotino | GR 3 IND N | M | 46-60 | - | - | - |
| Modern Era | Viale Sabotino | GR 4 IND B | F | 31-45 | - | - | 28.57 |
| Modern Era | Viale Sabotino | GR 4 IND D | F | 16-20 | - | - | 50.00 |
| Modern Era | Viale Sabotino | GR 4 IND F | F | 31-45 | 26.29 | 16.67 | 62.50 |
| Modern Era | Viale Sabotino | GR 4 IND H | M | 31-45 | - | - | 23.53 |
| Modern Era | Viale Sabotino | GR 4 IND I | M | 46-60 | - | - | 26.67 |
| Modern Era | Viale Sabotino | GR 4 IND L | M | 31-45 | - | - | 27.27 |
| Modern Era | Viale Sabotino | GR 4 IND M | M | 31-45 | - | - | 17.65 |
| Modern Era | Viale Sabotino | GR 4 IND O | F | 31-45 | - | - | 66.67 |
| Modern Era | Viale Sabotino | GR 4 IND S | F | 31-45 | - | - | 60.00 |
| Modern Era | Viale Sabotino | GR 5 IND A | F | 21-30 | - | - | 50.00 |
| Modern Era | Viale Sabotino | GR 5 IND E | M | 46-60 | - | - | 60.00 |
| Modern Era | Viale Sabotino | GR 6 IND F | F | 31-45 | - | - | 30.00 |
| Modern Era | Viale Sabotino | GR 6 IND I | M | 46-60 | - | - | 50.00 |
| Modern Era | Viale Sabotino | GR 6 IND L | M | 16-20 | 21.43 | 16.67 | 26.32 |
| Modern Era | Viale Sabotino | GR 7 IND C | F | 16-20 | - | - | 57.14 |
| Modern Era | Viale Sabotino | GR 7 IND E | F | 31-45 | - | 33.33 | 40.00 |
| Modern Era | Viale Sabotino | GR 9 IND A | F | 21-30 | - | - | 35.29 |
| Contemporary Era | Collezione Cimiteriale | 7 | F | 61-80 | 35.71 | 33.33 | 25.00 |
| Contemporary Era | Collezione Cimiteriale | 10 | M | 61-80 | - | 16.67 | 25.00 |
| Contemporary Era | Collezione Cimiteriale | 20 | F | >80 | - | - | 31.25 |
| Contemporary Era | Collezione Cimiteriale | 21 | F | >80 | - | - | 11.76 |
| Contemporary Era | Collezione Cimiteriale | 24 | M | 46-60 | - | - | 6.67 |
| Contemporary Era | Collezione Cimiteriale | 25 | M | 46-60 | 33.43 | 50.00 | 23.81 |
| Contemporary Era | Collezione Cimiteriale | 26 | M | 61-80 | - | - | 56.25 |
| Contemporary Era | Collezione Cimiteriale | 27 | M | 21-30 | 35.71 | 50.00 | 33.33 |
| Contemporary Era | Collezione Cimiteriale | 31 | F | 61-80 | - | - | 23.53 |
| Contemporary Era | Collezione Cimiteriale | 32 | F | >80 | - | - | 23.53 |
| Contemporary Era | Collezione Cimiteriale | 35 | M | >80 | - | - | 29.41 |
| Contemporary Era | Collezione Cimiteriale | 37 | F | 61-80 | - | - | 29.41 |
| Contemporary Era | Collezione Cimiteriale | 39 | M | 61-80 | - | - | 5.88 |
| Contemporary Era | Collezione Cimiteriale | 63 | M | >80 | - | - | 17.65 |
| Contemporary Era | Collezione Cimiteriale | 65 | M | 61-80 | - | - | 35.29 |
| Contemporary Era | Collezione Cimiteriale | 68 | F | >80 | - | - | 35.29 |
| Contemporary Era | Collezione Cimiteriale | 69 | M | 61-80 | - | - | 23.53 |
| Contemporary Era | Collezione Cimiteriale | 82 | M | 31-45 | 0.00 | 0.00 | 9.52 |
| Contemporary Era | Collezione Cimiteriale | 88 | F | >80 | - | 50.00 | 23.53 |
| Contemporary Era | Collezione Cimiteriale | 92 | M | 61-80 | 14.29 | 33.33 | 11.11 |
| Contemporary Era | Collezione Cimiteriale | 108 | F | 61-80 | - | - | 6.67 |
| Contemporary Era | Collezione Cimiteriale | 143 | M | 61-80 | 25.00 | 50.00 | 42.11 |
| Contemporary Era | Collezione Cimiteriale | 156 | F | 61-80 | - | - | 23.53 |
| Contemporary Era | Collezione Cimiteriale | 166 | M | >80 | - | - | 17.65 |
| Contemporary Era | Collezione Cimiteriale | 169 | F | >80 | - | - | 23.53 |
| Contemporary Era | Collezione Cimiteriale | 185 | M | 21-30 | 33.43 | 33.33 | 23.81 |
| Contemporary Era | Collezione Cimiteriale | 200 | F | >80 | 21.43 | 50.00 | 19.05 |
| Contemporary Era | Collezione Cimiteriale | 277 | F | 61-80 | 31.00 | 66.67 | 47.62 |
| Contemporary Era | Collezione Cimiteriale | 289 | M | 31-45 | - | - | 13.33 |
| Contemporary Era | Collezione Cimiteriale | 336 | M | 61-80 | - | - | 17.65 |
| Contemporary Era | Collezione Cimiteriale | 338 | F | >80 | - | - | 17.65 |
| Contemporary Era | Collezione Cimiteriale | 347 | M | 31-45 | - | - | 29.41 |
| Contemporary Era | Collezione Cimiteriale | 351 | F | 46-60 | 4.86 | 16.67 | 23.81 |
| Contemporary Era | Collezione Cimiteriale | 365 | M | 46-60 | 20.29 | 66.67 | 42.86 |
| Contemporary Era | Collezione Cimiteriale | 366 | M | >80 | - | - | 23.53 |
| Contemporary Era | Collezione Cimiteriale | 395 | M | 61-80 | - | - | 22.22 |
| Contemporary Era | Collezione Cimiteriale | 407 | F | >80 | 14.29 | 50.00 | 14.29 |
| Contemporary Era | Collezione Cimiteriale | 409 | F | 21-30 | 26.29 | 0.00 | 18.75 |
| Contemporary Era | Collezione Cimiteriale | 423 | F | 61-80 | 28.57 | 50.00 | 22.22 |
| Contemporary Era | Collezione Cimiteriale | 426 | F | 61-80 | - | - | 23.53 |
| Contemporary Era | Collezione Cimiteriale | 442 | F | 61-80 | 25.00 | 66.67 | 25.00 |
| Contemporary Era | Collezione Cimiteriale | 481 | F | >80 | 28.57 | 33.33 | 28.57 |
| Contemporary Era | Collezione Cimiteriale | 499 | F | >80 | - | - | 28.57 |
| Contemporary Era | Collezione Cimiteriale | 857 | M | 21-30 | 12.00 | 66.67 | 40.00 |
| Contemporary Era | Collezione Cimiteriale | 897 | M | 46-60 | - | - | 23.53 |
| Contemporary Era | Collezione Cimiteriale | 948 | M | 61-80 | 28.57 | 66.67 | 28.57 |
| Contemporary Era | Collezione Cimiteriale | 1007 | F | >80 | - | - | 28.57 |
| Contemporary Era | Collezione Cimiteriale | 1200 | F | 61-80 | - | - | 29.41 |
| Contemporary Era | Collezione Cimiteriale | 1213 | F | 21-30 | 12.00 | 33.33 | 21.05 |
| Contemporary Era | Collezione Cimiteriale | 1217 | M | 46-60 | 4.86 | 16.67 | 4.76 |
